# Ghost cell glaucoma after intravitreous injection of ranibizumab in proliferative diabetic retinopathy

**DOI:** 10.1186/s12886-020-01422-z

**Published:** 2020-04-15

**Authors:** Jun Xu, Meng Zhao, Ji peng Li, Ning pu Liu

**Affiliations:** Address: Beijing Tongren Eye Center, Beijing Key Laboratory of Ophthalmology and Visual Science, Beijing Tongren Hospital, Capital Medical University, No1. Dongjiaominxiang street, Dongcheng District, Beijing, 100730 China

**Keywords:** Intravitreous injection, Vitrectomy, Proliferative diabetic retinopathy, Ghost cell glaucoma

## Abstract

**Background:**

The development of ghost cell glaucoma in patients with proliferative diabetic retinopathy (PDR) after intravitreous injection (IV) was rare. Here we reported a series of patients with PDR who received Intravitreous Ranibizumab (IVR) and developed ghost cell glaucoma and analyzed the potential factors that might be related to the development of ghost cell glaucoma.

**Methods:**

Retrospective case series study. The medical records of 71 consecutive eyes of 68 PDR patients who received vitrectomy after IVR from January 2015 to January 2017 were reviewed. The development of ghost cell glaucoma after IVR was recorded. Characteristics of enrolled patients were retrieved from their medical charts. Factors associated with ghost cell glaucoma were compared between eyes with the development of ghost cell glaucoma and eyes without the development of ghost cell glaucoma. Variables were further enrolled in a binary backward stepwise logistic regression model, and the model that had the lowest AIC was chosen.

**Results:**

There were 8 out of 71 eyes of the PDR patients developed ghost cell glaucoma after they received IVR. The interval between detection of elevation of intraocular pressure (IOP) and IV ranged from 0 to 2 days. Among them, after IVR, there were two eyes had IOP greater than 30 mmHg within 30 min, four eyes showed normal IOP at 30 min, and then developed ghost cell glaucoma within 1 day, two eyes developed ghost cell glaucoma between 24 and 48 h. The mean IOP was 46.5 ± 8.0 mmHg. All patients gained normal IOP after vitrectomy without medicine for lowering IOP. The presence of ghost cell glaucoma was associated with tractional retinal detachment (RR = 4.60 [2.02 ~ 8.48], *p* = 0.004) and fibrovascular membrane involving disk (RR = -3.57 [− 7.59 ~ − 0.92], *p* = 0.03) (AIC = 39.23, AUC = 0.88) in a logistic regression model.

**Conclusion:**

Attention to postoperative IOP should be paid to patients with PDR undergoing vitrectomy who receive a preoperative IV of anti-VEGF agents. PDR patients with tractional retinal detachment or fibrovasucular membrane involving optic disc are more likely to develop ghost cell glaucoma after IV.

## Background

Ghost cell glaucoma can occur in patients with long-standing vitreous hemorrhage (VH) [1, 2] and can be refractory to lower intraocular pressure medications. It can cause irreversible visual impairment [3]. It has been reported that ghost cell glaucoma generally occurs where there are VH and disruption of the anterior hyaloid surface following surgery or trauma [3–6].

Severe vision impairments caused by proliferative diabetic retinopathy (PDR) often result from complications such as neovascularization and fibrovascular proliferation [1]. Non-clearing VH, TRD, extensive fibrovascular proliferation are common indications of vitrectomy in PDR patients [2, 3]. Intravitreous injection (IV) of anti-vascular endothelial growth factor (VEGF) agents can be used as an adjunctive method to vitrectomy in eyes with VH due to PDR [4–8]. It has been proved to be a safe and effective method to shorten the overall surgery time, lower the rate of intraoperative complications, and reduce the occurrence of postoperative hemorrhage [9, 10]. It is reported that the development of ghost cell glaucoma in PDR patients who received IV is rare [7–10].

At present, IV of anti-VEGF agents has also been proved to be safe and effective in the treatment of age-related macular degeneration [11], cystoid macular edema due to retinal vein occlusion [12] in large clinical trials. The sustained intraocular pressure elevation was reported to be related to the total number of injections, a greater frequency of injection, and pre-existing glaucoma [13]. The occurrence of ghost cell glaucoma after IV was rarely reported in patients with either age-related macular degeneration or retinal vein occlusion who need IV [11, 12].

Here we reported a series of patients with PDR who developed ghost cell glaucoma after pre-vitrectomy IV and analyzed the potential factors that might be related to the development of ghost cell glaucoma.

## Methods

This study was a retrospective case series of consecutive patients undergoing intravitreous injection of ranibizumab (IVR) with a diagnosis of PDR who were planned to take vitrectomy after IV. Records of 126 patients were retrospectively reviewed in the study from January 2015 to January 2017 in the Beijing Tongren Eye Center. This study was approved by the Ethics Committee of Beijing Tongren Hospital and adhered to the tenets of the Declaration of Helsinki.

Inclusion criteria: 1) Patients diagnosed with PDR, 2) patients took an IVR before vitrectomy, 3) Records with intraocular pressure (IOP) values measured before and after IVR. Exclusion criteria: 1) patients failed to finish at least 1-month follow-up after vitrectomy; 2) patients with a history of pre-existed open-angle glaucoma, 3) patients with pre-existed narrow/closed angle, 4) patients received an intravitreous or subtenon injection of corticosteroids or steroid eye drops within the latest six months, 5) uncontrolled neovascular glaucoma by at least 3 kinds of antiglaucoma medicines.

All patients underwent comprehensive ophthalmological examinations, including best-corrected visual acuity (BCVA) testing using a decimal VA chart, slit-lamp biomicroscopy, IOP measurement, dilated fundus examination with indirect ophthalmoscopy, color fundus photograph, optical biometry, optic coherent tomography (OCT), B scan. Gonioscopy was considered when iris neovascularization was found. BCVA, the axial length, the presence of posterior vitreous detachment (PVD, was defined as the presence of a Weiss ring and visible posterior vitreous cortex under the slit-lamp biocular biomicroscopy examination by the same surgical doctor or by B scan [14], PVD was confirmed by findings in triamcinolone acetonide-assisted vitrectomy), history of diabetes mellitus, history of visual acuity decrease, use of insulin, history of retinal photocoagulation for diabetic retinopathy, sex, age, refraction, presence of iris neovascularization, intraocular lens (IOL), dense VH that obscured the view of the optic disc and details of the fundus, tractional retinal detachment that threatened the central vision or caused repeated VH, fibrovascular membrane involving the disk, presence of macular edema were recorded as the baseline data.

An IV of ranibizumab 0.5 mg was performed 1–10 days before vitrectomy. All injections were performed by one surgeon. All patients underwent a 3-port pars plana 23-gauge vitrectomy under general anesthesia. Phacoemulsification surgery was performed before vitrectomy in case of necessary determined by the surgeon. The presence of PVD, a tractional retinal detachment that threatened central vision, fibrovascular membrane involving the disk were confirmed in the vitrectomy after removal of dense VH and recorded. The silicon oil tamponade and laser points during vitrectomy were recorded.

All patients underwent a complete series of IOP measurements with an air tonometer (Nidek, Tonoref 3). IOP was measured before IVR, 30 min, 2 h, 1d, 2d, 3d after IV. If elevated IOP occurred, IOP was measured twice a day until the IOP was controlled. The ghost cell glaucoma was defined as the presence of both high IOP and ghost cells in the anterior chambe r[12]. Follow-up visits were scheduled at 1,2,7,14 and 30 days after the initial surgery. The examination included BCVA, IOP, slit lamp examination, dilated fundus examination.

Statistical analysis was performed using R version 3.20 (http://www.R-project.org). Patient characteristics were retrieved from their medical charts and recorded in Epidata EntryClient version 2.0.3.15 (http://epidata.dk). BCVA results were converted to a logMAR value for statistical analysis. Mean and standard deviation (SD) were calculated for continuous variables with a normal distribution. Median with quartiles was calculated for continuous variables with a non-normal distribution. The t-test or Mann-Whitney U test was carried out for continuous variables. The Chi-square test or Fisher’s exact test was carried out for discrete data. To explore the potential factors that might influence the occurrence of ghost cell glaucoma, we divided the patients into two groups, patients with ghost cell glaucoma and patients without ghost cell glaucoma after IVR. Several factors including duration of diabetes mellitus, the onset of decrease vision, use of insulin, pre-existence of pan-retinal photocoagulation (PRP) for DR, refraction error, axial length, sex, age, presence of PVD, presence of iris neovascularization (NVI), TRD that threatened the central vision or caused repeated VH, IOL, fibrovascular membrane involving the disk, presence of clinically significant macular edema were compared between two groups (Table 1). Variables (*p* < =0.4) were further enrolled in a binary backward stepwise logistic regression model. One variable was included or excluded from the model each time by comparing the Akaike information criterion (AIC) value, and the model that had the lowest AIC was chosen. The model was accessed by the receiver operating characteristic curve (ROC curve).

**Table 1:** The basic characteristics of patients with proliferative diabetic retinopathy received with an intravitreous injection of ranibizumab

Characteristics	Patients in all	Patients who developed ghost cell glaucoma	Patients failed to develop ghost cell glaucoma	Correlations with the presence of post-vitreous injection ghost cell glaucoma (*p*-value)
age (y, mean ± SD)	51.46 ± 11.26	47.85 ± 12.05	51.92 ± 11.11	0.34
gender (male, n, %)	41, 57.75%	5, 62.5%	36,57.1%	0.77
duration of diabetes mellitus (y, mean ± SD)	10.00 ± 6.69	9.2 ± 6.2	9.5 ± 6.8	0.92
onset of vision decrease (m, median, 1st Qu, 3rdQu)	5.38 ± 4.77	3.63 ± 2.13	5.60 ± 4.97	0.35 ^a^
use of insulin (y, n, %)	47, 66.20%	6, 75%	41,65.1%	0.57
history of retinal photocoagulation (y, n, %)	42, 59.15%	7, 87.5%	35, 55.5%	0.084 (7/42 vs 1/29 Laser point *p* < 0.001)
refraction error				0.67
myopia (n,%)	10, 14.1%	1,12.5%	9, 14.2%	
emmetropia (n,%)	57, 80.3%	6, 75.0%	51, 81%	
hyperopia (n,%)	4, 5.6%	1,12.5%	3, 4.8%	
presence of iris neovascular (n, %)	8, 11.3%	3, 37.5%	3, 4.8%	< 0.001
presence of dense vitreous hemorrhage (n, %)	70, 98.59%	8, 100%	62, 98.4%	0.72
presence of tractional retinal detachment (n, %)	58, 81.7%	3, 37.5%	55, 87.3%	< 0.001 Trd 11.3% non-trd 88.7
presence of PVD (n, %)	15, 21.1%	1, 12.5%	14, 22.2%	0.53
presence of fibromembrane involving disk (n, %)	40, 56.3%	6, 75%	34, 54%	0.25 11.3 vs 88.7
BCVA				0.72
LP ~ <=0.01 (n, %)	32, 45.07%	6, 75%	26, 41.3%	
0.01 ~ <=0.1 (n, %)	31, 43.66%	2, 25%	29, 46.0%	
0.1 ~ <=0.5 (n, %)	6, 8.45%	0	6, 9.5%	
> 0.5 (n, %)	2, 2.82%	0	2, 3.2%	
Pseudophakic eye (n, %)	3, 4.2%	1, 12.5%	2, 3.2%	0.22
axial length (mm, mean ± SD)	22.89 ± 0.75	23.09 ± 0.52	22.88 ± 0.78	0.46
silicon tamponade (n, %)	20, 28.2%	2, 25%	18, 28.6%	0.83
photocoagulation burns during vitrectomy (points, median, 1st Qu, 3rdQu)	1046, 535, 1382	660,303,1023	1074, 588, 1382	0.18
prescence of macular edema (n, %)	28, 39.4%	4, 50%	24, 38.1%	0.52
tractional retinal detachment involving macular (n, %)	16, 22.5%	1, 12.5%	15,23.8%	0.47
Interval between	3, 2,4	4,2,6	3,2,4	0.36
IVR and vitrectomy (d, median, 1st Qu, 3rdQu)				
<=2 d (n, %)	21, 29.58%	2, 25%	19, 30.2%	
2 ~ <=5d (n, %)	39, 54.93%	3, 37.5%	36, 57.1%	
>5d (n, %)	11, 15.49	3, 37.5%	8, 12.7%	

a Wilcoxon rank sum test

## Results

A total of 71 eyes of 68 patients were included. Among them, 3 patients received an IV in both eyes. The patients’ baseline characteristics are presented in Table 1.

There were 8 out of 71 eyes of the PDR patients developed ghost cell glaucoma after they received IVR. The basic characteristics of the patients developed ghost cell glaucoma were listed in Table 1. The interval between the onset of visual symptom and IV ranged from 0.2 m to 24 m, with a median of 4 m [1st quality was 2, 3rd quality was 7]. There were 7 out of 8 patients who failed to complete pan-retinal photocoagulation due to dense VH. Three patients were presented with tiny iris neovascularization on the pupil margin, and they had normal IOP before IV. Three patients were presented with dense VH and sight threatened tractional retinal detachment. The interval between detection of elevation of IOP and IV ranged from 0 to 2 days. Among them, there were 2 eyes had IOP higher than 30 mmHg within 30 min after IVR, 4 eyes showed normal IOP at 30 min after IVR, and then developed ghost cell glaucoma within 1 day after IVR, 2 eyes developed ghost cell glaucoma between 24 and 48 h after the IVR. The mean maximum IOP was 46.5 ± 8.0 mmHg. Five patients required methazolamide, brimonidine tartrate, and carteolol hydrochloride to control IOP, and three patients required additional paracentesis to control IOP. Three patients had persistent ghost cells in the anterior chamber after vitrectomy. All patients gained normal IOP without medication for lowering IOP after vitrectomy. All patients did not show vitrectomy related complications during follow-up.

**Fig. 1:**
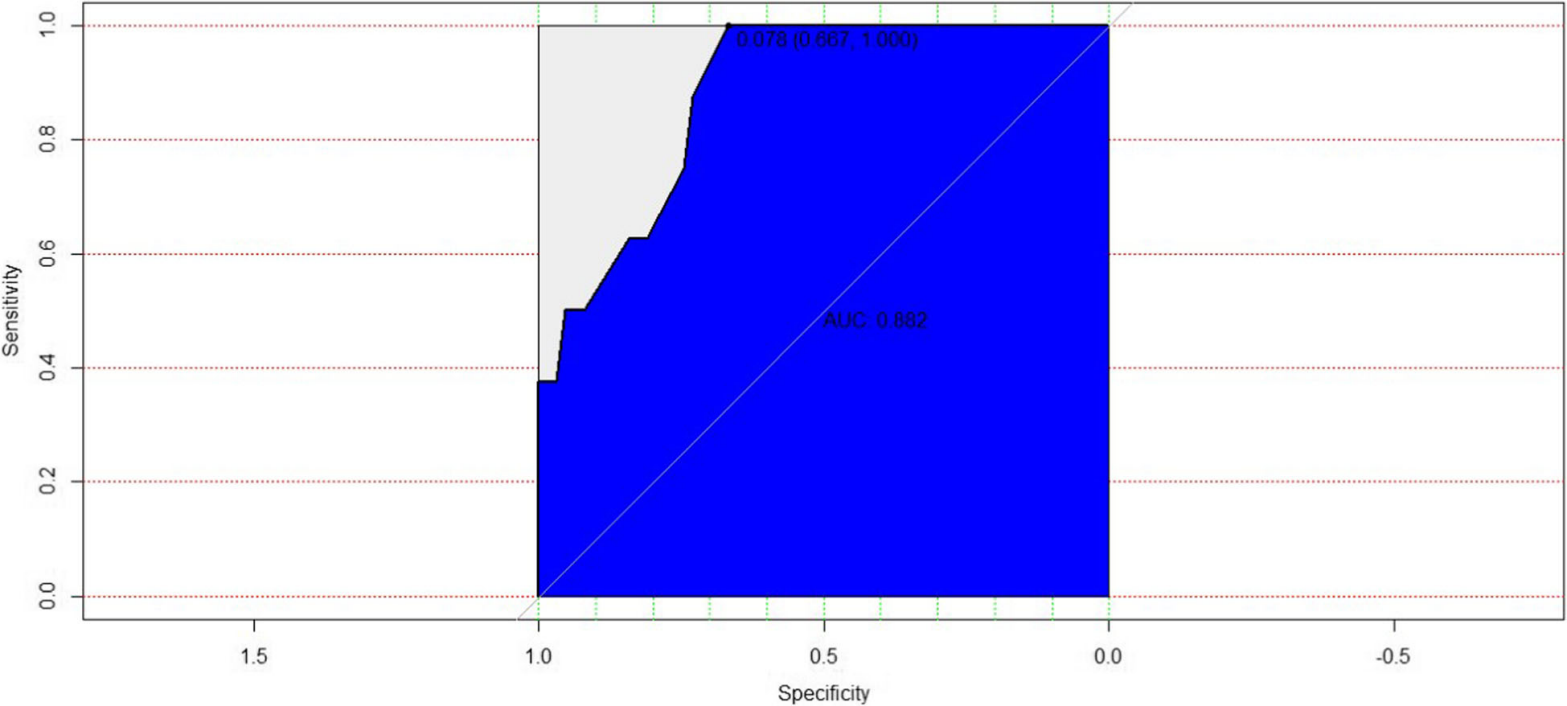
The receiver operating characteristic curve for the model of logistic regression analysis for potential factors associated with the development of ghost cell glaucoma

To confirm the potential factors that might be related to the development of ghost cell glaucoma, the data were divided into two groups by the presence of post-IVR ghost cell glaucoma. Variables in Table 1 with a *p*-value <= 0.4 in the two independent sample comparison were selected in the initial logistic regression model, including age, the onset of vision decrease, history of retinal photocoagulation, presence of iris neovascular, presence of TRD, presence of fibrovascular membrane involving disk, and pseudophakic eye. Variables were further enrolled in a binary backward stepwise logistic regression model. One variable was included or excluded from the model each time by comparing the Akaike information criterion (AIC) value, and the model that had the lowest AIC was chosen. The presence of ghost cell glaucoma was associated with tractional retinal detachment (RR = 4.60 [2.02 ~ 8.48], *p* = 0.004) and fibrovascular membranes involving disk (RR = -3.57 [− 7.59 ~ − 0.92], *p* = 0.03) (AIC = 39.23, AUC = 0.88). The logistic regression ROC curve was shown in Fig. 1.

## Discussion

Here we report a group of patients with PDRss who developed ghost cell glaucoma after IVR and showed some potential factors that may be related to the development of ghost cell glaucoma.

The ghost cell glaucoma is rare [7, 15] after IV in PDR patients, as reported. It has been said that sustained IOP elevation after IV caused by ghost cell glaucoma only occurs in 3% PDR eyes with VH [15], but do not occur in eyes with diabetic macular edema or PDR without VH [16, 17]. In our series of patients with PDR, the incidence of ghost cell glaucoma after IV (8/71) is higher than previously reported, which was published as 0.7–2.2% [7, 15, 18], but is much lower than eyes with PDR with post-vitrectomy VH [8]. In L. Liu et al. ‘s study [8], the incidence of ghost cell glaucoma in eyes with PDR after intravitreal bevacizumab for post-vitrectomy VH is 3 out of 8 eyes. Ghost cell glaucoma generally occurs where there are VH and disruption of the anterior hyaloid surface following surgery or trauma [3–6]. A higher incidence of ghost cell glaucoma after vitrectomy in L. Liu et al. ‘s study might suggest that the ghost cell could gain entrance to the anterior chamber more easily in the condition of the removal of vitreous by vitrectomy. In our series, the PDR patients had a long-standing or recurrent VH that required vitrectomy intervention. Although the anterior hyaloid surface of the vitreous body was relatively intact, a large amount of pre-existing ghost cell in the vitreous, the disturbance of fluid in the vitreous and transient changes of intraocular pressure during IV may push the ghost cell into the anterior chamber and contribute to the higher occurrence of ghost cell glaucoma in our study. Moreover, transient elevated pressure in vitreous may damage the anterior hyaloid surface of the vitreous body during IV. Besides, we had monitored the post-IVR IOP more frequently in our patients than which was recommended by IV guidelines [19] or previous studies [15, 18]. The frequent measurement of IOP after IVR might facilitate the early detection of ghost cell glaucoma. Our data indicated that the development of ghost cell glaucoma is more common than previously thought in PDR patients with severe VH who underwent IVR. More attention should be paid to a slower injection and maintain a relatively stable IOP during IV to lessen the development of ghost cell glaucoma. Monitoring of IOP after IV should be recommended in cases of PDR patients with severe VH.

The onset of ghost cell glaucoma after IV in our series is different from eyes without IV. It has been reported that the ghost cell can occur in the vitreous and enter the anterior chamber in phakic eyes spontaneously between 18 months and 4 years after VH [1, 2]. The development of ghost cell glaucoma within 1 week after IV of bevacizumab in eyes with postoperative VH after vitrectomy for PDR has also been reported [8]. In our study, the development of ghost cell glaucoma might be found right after IVR or 1–2 days delay. The development of ghost cell glaucoma was more rapidly in eyes with IV compared with eyes without IV [1]. It is suggested that IVR was a risk factor for the development of ghost cell glaucoma in eyes with VH. More attention should be paid on postoperative IOP on the first 2 days after IV in patients with PDR and VH.

We tried to identify the potentially related factors to the development of ghost cell glaucoma in PDR patients who received IVR before vitrectomy. Among the patients who developed ghost cell glaucoma, we found most of the patients failed to receive any PRP treatment, and some of them had iris neovascularization, which may indicate long-standing history and poorly controlled and severe ischemic retinal condition. We also found that tractional retinal detachment (RR = 4.60 [2.02 ~ 8.48], *p* = 0.004) and fibrovascular membranes involved optic disc (RR = -3.57 [− 7.59 ~ − 0.92], *p* = 0.03) are factors related to the development of ghost cell glaucoma. We failed to identify either NVI or lacking PRP as potential factors related to the development of ghost cell glaucoma in the logistics regression model. It should be provided by a further large sample study. The late complications of PDR result from the development of posterior vitreous detachment and contraction of the fibrovascular membranes [20, 21]. Both tractional retinal detachment and fibrovascular membrane on optic disk may result from adherent of vitreous to the retina, which provides a scaffold for retinal neovascularization to grow into vitreous and can cause repeated VH [16]. The latter one may be the source of a large amount of ghost cell in vitreous [1, 8] in our series. The higher incidence of ghost cell glaucoma in our series of PDR patients may also due to the high amount of ghost cells in the vitreous body caused by long-standing VH or repeated VH.

Vitrectomy is effective as a treatment to lowing IOP in the condition of ghost cell glaucoma by cleaning both the VH and reservoir of ghost cells in the vitreous as previously reported [3, 22]. In our study, All patients gained normal IOP after vitrectomy without further medications for lowering IOP.

We failed to show that the presence of VH is associated with the development of ghost cell glaucoma due to a small sample size of patients with VH without tractional retinal detachment. The previous study had shown that IVR would be used for the treatment of PDR with VH in eyes that do not always receive vitrectomy [15, 18]. The incidence of ghost cell glaucoma is less than 1% in patients with relatively newly developed VH due to PDR. However, the data on the first week after IV was laking [15]. Further study with a larger PDR sample with newly develop VH is required to confirm whether VH alone was related to the development of ghost cell glaucoma after IV. It may extend our recommendations on monitor IOP after IV from eyes planed to vitrectomy due to long-standing or recurrent VH to eyes with VH receiving anti-VEGF injections as monotherapy.

The limitation of this study was due to it was a retrospective case series. We failed to include enough samples with pseudophakic eye or VH without tractional retinal detachment. The selected bias was also presented due to the patients enrolled in this study were in a tertiary hospital. More complicated cases with longer duration may be enrolled in this study, which may contribute to the high occurrence of ghost cell glaucoma after IV.

## Conclusion

In summary, this study reports a series of patients with PDR who developed ghost cell glaucoma after pre-vitrectomy IV of ranibizumab. The development of ghost cell glaucoma varies from 0 to 2 days and is most anticipated within 1 day after IV. The presence of tractional retinal detachment and fibrovascular membrane on the optic disc are factors may be related to the development of ghost cell glaucoma after IV. Attention on postoperative IOP should be paid to patients with PDR undergoing vitrectomy who receive a preoperative IV of anti-VEGF agents, especially in patients with severe PDR.

## Data Availability

The datasets used and/or analysed during the current study are available from the corresponding author on reasonable request.

## Abbreviations

AIC: Akaike information criterion
BCVA: Best-corrected visual acuity
FA: Fluorescence angiography
IOP: Intraocular pressure
IV: Intravitreous injection
IVR: Intravitreous injection of ranibizumab
IOL: Intraocular lens
NVI: Iris neovascularization
OCT: Optical coherence tomography
PVD: Posterior vitreous detachment
ROC curve: Receiver operating characteristic curve
SD: Standard deviation
